# Promastigote secretory gel from natural and unnatural sand fly vectors exacerbate *Leishmania major* and *Leishmania tropica* cutaneous leishmaniasis in mice

**DOI:** 10.1017/S0031182019001069

**Published:** 2019-08-29

**Authors:** E. Giraud, M. Svobodová, I. Müller, P. Volf, M. E. Rogers

**Affiliations:** 1 Faculty of Infectious Tropical Diseases, Department of Immunology and Infection, London School of Hygiene and Tropical Medicine, London, UK; 2 Department of Parasitology, Faculty of Science, Charles University in Prague, Prague, Czech Republic; 3 Faculty of Medicine, Division of Infectious Diseases, Department of Medicine, Section of Immunology at St. Mary's, Imperial College London, London, UK; 4 Faculty of Infectious Tropical Diseases, Department of Disease Control, London School of Hygiene and Tropical Medicine, London, UK

**Keywords:** Cutaneous leishmaniasis, *Leishmania major*, *Leishmania mexicana*, *Leishmania tropica*, *Leishmania*, PSG, sand fly, transmission, zoonoses

## Abstract

*Leishmania* rely heavily on glycans to complete their digenetic life cycle in both mammalian and phlebotomine sand fly hosts. *Leishmania* promastigotes secrete a proteophosphoglycan-rich gel (Promastigote Secretory Gel, PSG) that is regurgitated during transmission and can exacerbate infection in the skin. Here we explored the role of PSG from natural *Leishmania*-sand fly vector combinations by obtaining PSG from *Leishmania (L.) major*-infected *Phlebotomus (P.) papatasi* and *P. duboscqi* and *L. tropica*-infected *P. arabicus*. We found that, in addition to the vector's saliva, the PSG from *L. major* and *L. tropica* potently exacerbated cutaneous infection in BALB/c mice, improved the probability of developing a patent cutaneous lesion, parasite growth and the evolution of the lesion. Of note, the presence of PSG in the inoculum more than halved the prepatent period of cutaneous *L. tropica* infection from an average of 32 weeks to 13 weeks. In addition, *L. major* and *L. tropica* PSG extracted from the permissive experimental vector, *Lutzomyia (Lu.) longipalpis*, also exacerbated infections in mice. These results reinforce and extend the hypothesis that PSG is an important and evolutionarily conserved component of *Leishmania* infection that can be used to facilitate experimental infection for drug and vaccine screening.

## Introduction

*Leishmania* are parasitic protozoa, which are transmitted by the bite of a female phlebotomine sand fly. Depending on the species of parasite, *Leishmania* can cause a spectrum of diseases, ranging from painful and disfiguring skin lesions (cutaneous leishmaniasis, CL, and mucocutaneous leishmaniasis) to fatal visceral infection of the spleen and liver (visceral leishmaniasis). In the mammalian host, *Leishmania* parasites reside within professional phagocytic cells of the monocyte-macrophage lineage as amastigote forms. When they are taken up by a sand fly they multiply and undergo a series of transformations as various flagellated promastigote forms; culminating in the differentiation to a mammal-infective stage, the metacyclic promastigote (reviewed in Bates, 1997).

It is becoming increasingly clear that transmission by sand fly bite has considerable bearing on the infection and is vastly more efficient than needle-based infection of parasites alone (reviewed by Serafim *et al*., 2017). From a sand fly bite metacyclic promastigotes are delivered into the skin of a host in the context of a unique inflammatory environment (Belkaid *et al*., 1998; Kamhawi *et al*., 2000). This is generated by the complex combination of parasite dose (Kimblin *et al*., 2008), dose composition (i.e. proportion of metacyclic and non-metacyclic promastigotes) (Giraud *et al*., 2019), damage caused by the bite (Giraud *et al*., 2018), sand fly-associated bacteria (Dey *et al*., 2018; Giraud *et al*., 2019) and the introduction of the vector saliva and other parasite-released molecules (Titus and Ribeiro, 1988; Belkaid *et al*., 1998; Rogers *et al*., 2004; Volfova *et al*., 2008; Atayde *et al*., 2015).

In the sand fly, *Leishmania* secrete high molecular weight proteophosphoglycans [filamentous proteophosphoglycan, fPPG; secreted acid phosphatase and a number of poorly defined lower molecular weight proteophosphoglycans (Montgomery *et al*., 2002)], which condense in the limited volume of the sand fly anterior midgut to form a gel-like obstruction, termed the Promastigote Secretory Gel (PSG) (Ilg *et al*., 1996; Stierhof *et al*., 1999; Rogers *et al*., 2002, 2004). PSG, has been noted for many natural and experimental *Leishmania*-sand fly combinations (reviewed by Rogers, 2012) and the blocked fly phenotype has been hypothesized to contribute to the transmission of leishmaniasis by encouraging the regurgitation of parasites (Shortt and Swaminath, 1928). The role of PSG was first demonstrated in 2004 using the experimental, yet highly transmissible, combination of *Leishmania mexicana* and *Lutzomyia longipalpis* (Rogers *et al*., 2004). Blocked sand flies were shown to regurgitate an average of 1000 parasites and a proportion of the PSG during transmission. Co-inoculation of this dose of *L. mexicana* metacyclic promastigotes with PSG promoted the survival and growth of amastigotes, which strongly aggravated skin infections in mice (Rogers *et al*., 2004, 2009). Using this sand fly in a natural parasite-vector combination, PSG was also shown to exacerbate cutaneous and visceral infection of *Leishmania infantum* in mice (Rogers *et al*., 2010).

As it appears that PSG can manipulate both the sand fly and mammalian hosts for efficient transmission, we aimed to see if this was relevant to other natural models of leishmaniasis. Therefore, we recovered, characterized and tested the infection-modulating properties of PSG from a variety of *Leishmania*-sand fly combinations responsible for CL in the field.

## Materials and methods

### Infection of sand flies

*Leishmania major* strain FV39 clone 5 (RHO/SU/59/P) and *Leishmania tropica* from Sanilurfa, Turkey (MHOM/1999/TR/SU23) were originally isolated from human cutaneous lesions and have been routinely passaged through mice. Promastigotes of these parasites were cultured as previously described (Rogers *et al*., 2002), resulting in an enriched population of metacyclic promastigotes as determined by morphological analysis of Giemsa-stained parasites. Briefly, 1 × 10^6^ BALB/c lesion amastigotes per mL were transformed into promastigotes in M199 culture medium (Hank's modified with L-glutamine) supplemented with 10% heat-inactivated foetal calf serum (FCS, v/v), 1 × Basal Medium Eagle vitamins (v/v), 1% penicillin-streptomycin (v/v), 400 mm sodium bicarbonate (all Sigma UK), pH 7.2, at 26 °C for 3 days. Following this, promastigotes were sub-passaged to 5 × 10^5^ mL in fresh M199 promastigote medium and allowed to undergo metacyclogenesis by 10–13 days post-culture. The cultures were assessed daily to select those with the highest proportion of metacyclic promastigotes. Using this culture method typically yielded 80–85% metacyclic promastigotes. Five-day-old female sand flies were infected with amastigotes through an artificial membrane feeding system at 2 × 10^6^ amastigotes per mL in fresh rabbit blood and maintained in a 12 h light:dark cycle at 28 °C, 80% relative humidity with free access to 12.5% sucrose.

### Preparation of vector saliva and parasite PSG

Sand fly saliva was obtained from 6–8 day-old female uninfected flies by piercing individual salivary glands in ice-cold PBS, followed by centrifugation at 3000 rpm for 10 min to remove any salivary gland epithelia or debris (Rogers *et al*., 2004). Pools of 10 flies were processed at a time to prevent degradation of the saliva and a minimum of 60 flies processed to supply saliva for the co-infections. For *L. mexicana* and *L. infantum* we have observed that PSG accumulates within the *Lu. longipalpis* midgut from day 7 onwards (Rogers and Bates, 2007). Therefore, PSG plugs were isolated from the midguts of day 10–13 infected flies in PBS to ensure that all parasites had reached maturity i.e. completed metacyclogenesis. PSG was obtained by dissecting the gut along its length and removing the PSG plug manually using 30 gauge insulin syringe needles. Each PSG plug was transferred to a tube in 5 *µ*L of the dissection medium. Parasites were removed by centrifuging twice at 3000 rpm for 10 min followed by two spins at 7,000 rpm for 10 min. The average yield of PSG for each sand fly-*Leishmania* combination used is presented in Table 1. Saliva and PSG were filter-sterilized (0.2 *µ*m pore diameter) and stored in aliquots at −40 °C until use. The protein content of PSG and saliva was determined using the BCA method and standardized to 1 *µ*g per injection. As a negative control for the Western Blot, the luminal content of 30 age-matched, bloodfed uninfected flies was sampled by dissecting each gut in the same way as an infected fly then sucking 5 *µ*L of dissection medium near the incision. These were pooled and processed as above.

**Table 1.** The average amount of PSG recovered from sand flies used in this study

Sand fly species	Yield of PSG recovered from late-stage sand fly infections (average ± s.d. *μ*g/fly total protein)	
	*L. major*	*L. tropica*
*P. papatasi*	0.32 ± 0.13	nd: incompatible
*P. duboscqi*	0.502 ± 0.11	nd: incompatible
*P. arabicus*	nd: incompatible	0.36 ± 0.22
*Lu. longipalpis*	0.56 ± 0.29	0.38 ± 0.25

*P. papatasi* and *P. duboscqi* were infected with *L. major* and PSG extracted from day 12–13 p.i. *P. arabicus* was infected with *L. tropica* and PSG was extracted 12–13 p.i. *Lu. longipalpis* was infected with *L. major* and *L. tropica* and the PSG extracted 8 days p.i. For reference, *Lu. longipalpis* infected with *L. mexicana* yielded an average 0.88 ± 0.14 *µ*g protein per infected fly at 8–9 days p.i. nd, not done.

### Western blotting

PSG was separated by SDS PAGE using a 10% acrylamide/bis-acrylamide gel (Sigma) with an extended stacking gel (4% acrylamide/bis-acrylamide) as previously described (Rogers *et al*., 2004). Gels were transferred to activated nitrocellulose and probed with the LT15 (1:3000) monoclonal antibody against phosphoglycan disaccharide repeats [PO_4_-6Gal*β*1-4Man*α*1-]x (Ilg *et al*., 1996). Blots were developed using a biotinylated anti-mouse IgG secondary antibody and HRP immunoprecipitation (ABC Elite, VIP, Vector Labs).

### Infection of mice

Anaesthetized, 20 g, 6–8 week old female BALB/c mice were infected either by the intradermal injection of 1 × 10^3^ *L. major* into the dorsal surface of the footpad, or 1 × 10^6^ *L. tropica* metacyclic promastigotes into the shaved rump, in a total volume of 20 *µ*L. Under restraint, Vernier callipers were used to measure thickness of infected and uninfected footpads of mice to calculate lesion swelling (*L. major*), or the average diameter of rump lesions to calculate their area (*L. tropica*). At the end of experiments, mice were euthanized allowing parasite burdens to be determined. Burdens were determined by direct counting of amastigotes from footpad homogenates. All procedures involving animals were approved by a local Animal Welfare Committee and performed in accordance with the United Kingdom Government (Home Office), Czech Ministry of Health and EC regulations.

## Results

### PSG from *L. major*, *L. tropica* and *L. mexicana* bear similar Gal(*β*1-4)Man(*α*1)-PO_4_ containing glycans

**Fig. 1.**
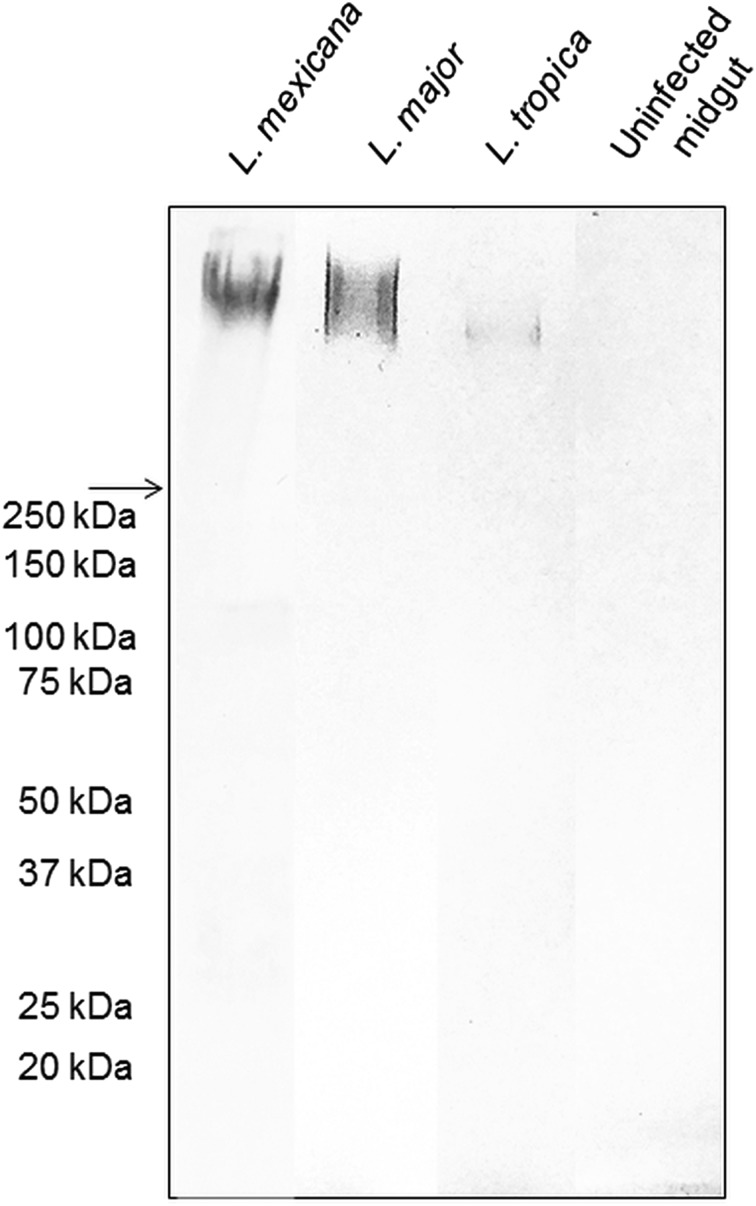
The composition of *Leishmania* glycans within PSG from the permissive vector *Lu. longipalpis*. Western Blot analysis of *L. major*, *L. tropica* and *L. mexicana* PSG probed with LT15, a mAb which recognizes the Gal-P-Man disaccharide backbone of all *Leishmania* phosphoglycans (Ilg *et al*., 1996). Each lane represents the equivalent of one sand fly's PSG extracted from mature infections (*L. major* and *L. tropica*, day 12–13 p.i.; *L. mexicana* day 8 p.i.). The last lane represents the luminal content of one uninfected sand fly midgut probed with LT15. Arrow indicates a junction between stacking and resolving gel. MWM, molecular weight markers.

The backbone of many O/N-linked *Leishmania* oligosaccharides contains phosphate-linked galactose-mannose disaccharide (Gal-Man-P) repeats (Ilg, 2000). Using Western blotting, the PSG from *L. major* and *L. tropica* extracted from the experimental vector *Lu. longipalpis*, were probed for Gal(*β*1-4)Man(*α*1)-PO_4_ repeating epitopes (Fig. 1). The absence of a reaction with the contents of uninfected midguts confirmed the specificity of the antibody for parasite glycans. All infected flies yielded PSGs containing a very high molecular weight smear retained in the 4% stacking gel that is likely to be the fPPG fraction. This gives the PSG much of its structure as a 3D matrix (Stierhoff *et al*., 1999) and confers the disease-exacerbating properties of PSG for *L. mexicana* (Rogers *et al*., 2004). In the experimental vector, *Lu. longipalpis*, the amount of fPPG was similar for *L. mexicana* and *L. major*, which were greater than the amount of fPPG produced by *L. tropica*. Notably, this is an experimental model so the precise amounts of fPPG in PSG from natural vectors may vary lipophosphoglycan (LPG) also contains Gal-Man-P repeats, however, based on the expected molecular weight (30–50 kDa for non-metacyclic and 70–90 kDa for metacyclic promastigotes), LPG was not detected in any of the PSG preparations.

### *L. major* PSG from its natural vectors, Phlebotomus (Phlebotomus) papatasi and Phlebotomus (Phlebotomus) duboscqi, exacerbates cutaneous infections *in vivo*

**Fig. 2.**
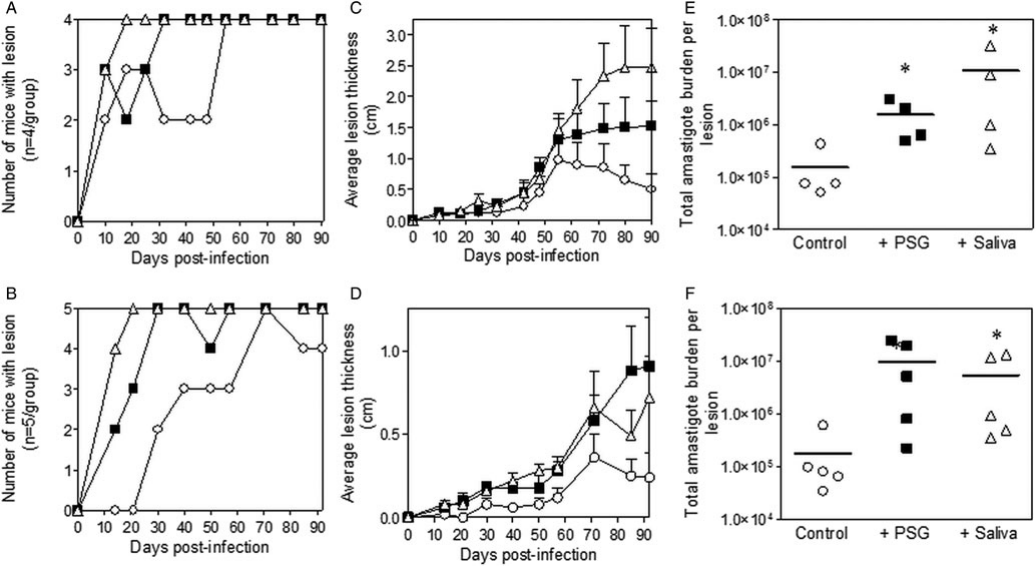
*L. major* infection in mice is exacerbated by the presence of PSG and saliva from natural vectors. *L. major* PSG was obtained from infected *P. papatasi* and *P. duboscqi* sand flies and saliva collected from uninfected flies (*P. papatasi:* A, C and E; *P. duboscqi*: B, D and F). One thousand *L. major* metacyclic promastigotes were injected i.d. into the footpads of BALB/c mice without (open circles) or with 1 *µ*g sterile PSG (closed squares) or 1 *µ*g sterile saliva (open triangles). (A and B) Proportion of mice presenting with cutaneous lesions over the course of study. (C and D) Footpad lesion evolution showing average lesion thickness + s.e.m. (E and F) Final parasite burdens of lesions from footpad homogenates. Each point represents individual mice and bars represent the average value per group. Infections performed in triplicate and representative data is shown, *n* = 4–5 mice per group. *: *P* < 0.05; **: *P* < 0.005 by Mann Whitney unpaired two-tailed *t*-test.

Inclusion of sterile *L. major* PSG from the natural vectors, *P. papatasi* or *P. duboscqi* promoted *L. major* infection in the skin of BALB/c mice (Fig. 2). The presence of *L. major* PSG during infection resulted in more mice developing a lesion earlier than the controls [10 *vs* 25 days for infections with *L. major* PSG from *P. papatasi* (Fig. 2A and C) and 14 *vs* 28 days for infections with *L. major* PSG from *P. duboscqi* (Fig. 2B and D)]. These infections grew faster than the controls, resulting in significantly larger lesions, which persisted (Fig. 2C and D). The final parasite burdens for these lesions showed that PSG benefited *L. major* growth, resulting in an average 30-fold higher number amastigotes per lesion compared to saline with metacyclics alone [10-fold for lesions with *L. major* PSG from *P. papatasi* (Fig. 2E), and 50-fold for *L. major* lesions co-inoculated with PSG from *P. duboscqi* (Fig. 2F)]. Similarly, the saliva from these two natural sand fly vectors significantly exacerbated infection with *L. major*, resulting in an average 45- to 60-fold higher amastigote burden at the end of the experiment.

### *L. tropica* PSG from its natural vector, *Phlebotomus (Adlerius) arabicus*, exacerbates cutaneous infection *in vivo*

**Fig. 3.**
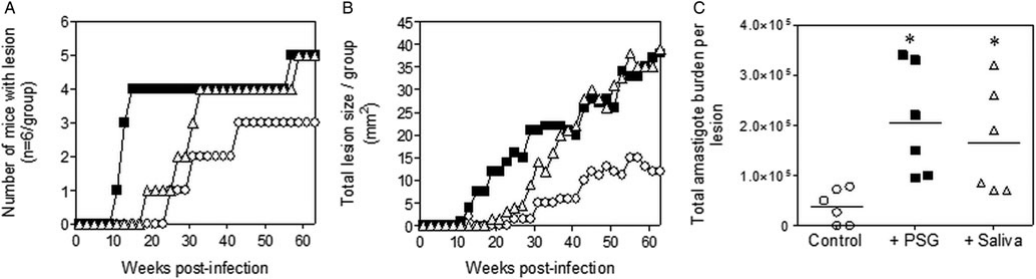
*L. tropica* infection in mice is exacerbated by PSG and saliva from a natural vector. *L. tropica* PSG was obtained from infected *P. arabicus* sand flies and saliva collected from uninfected flies. One million *L. tropica* metacyclic promastigotes were injected i.d. into the rumps of BALB/c mice without (open circles) or with 1 *µ*g sterile PSG (closed squares) or 1 *µ*g sterile saliva (open triangles). (A) Proportion of mice presenting with cutaneous lesions over the course of study. (B) Rump lesion evolution showing total lesion size. (C) Final parasite burdens of lesions from homogenates. Each point represents individual mice. Infections performed in duplicate and representative data is shown, *n* = 6 mice per group. *: *P* < 0.05 by Mann Whitney unpaired two-tailed *t*-test.

The infections with *L. tropica* significantly benefited from the presence of PSG, recovered from its natural vector, *P. arabicus* (Fig. 3). Even at a high dose of metacyclic promastigotes (1 × 10^6^), infection of mice with *L. tropica* in the absence of vector components took 30–33 weeks to generate cutaneous lesions (Fig. 3A and B). The presence of PSG cut this time by 60%, allowing the detection of lesions on the rumps of BALB/c mice by 13 weeks post-infection. The addition of PSG or saliva resulted in lesions that were chronic and non-healing, compared to the control infections that appeared to plateau in size after 43 weeks post-infection (Fig. 3B). Lesions from *L. tropica*-PSG co-infections grew significantly faster than the controls or those co-inoculated with *P. arabicus* saliva, resulting in an average 22-fold higher final parasite burdens compared to the controls. By comparison, co-infection with *P. arabicus* saliva resulted in an average 16-fold higher amastigote burden at the end of the experiment (Fig. 3B and C). Similarly, the proportion of mice, which developed lesions were equally high in the PSG and saliva co-infection groups (PSG: 83%, saliva: 83%, control: 50%) (Fig. 3A).

### *L. major* and *L. tropica* PSG from the experimental vector, *Lu. longipalpis*, exacerbates cutaneous infection *in vivo*

**Fig. 4.**
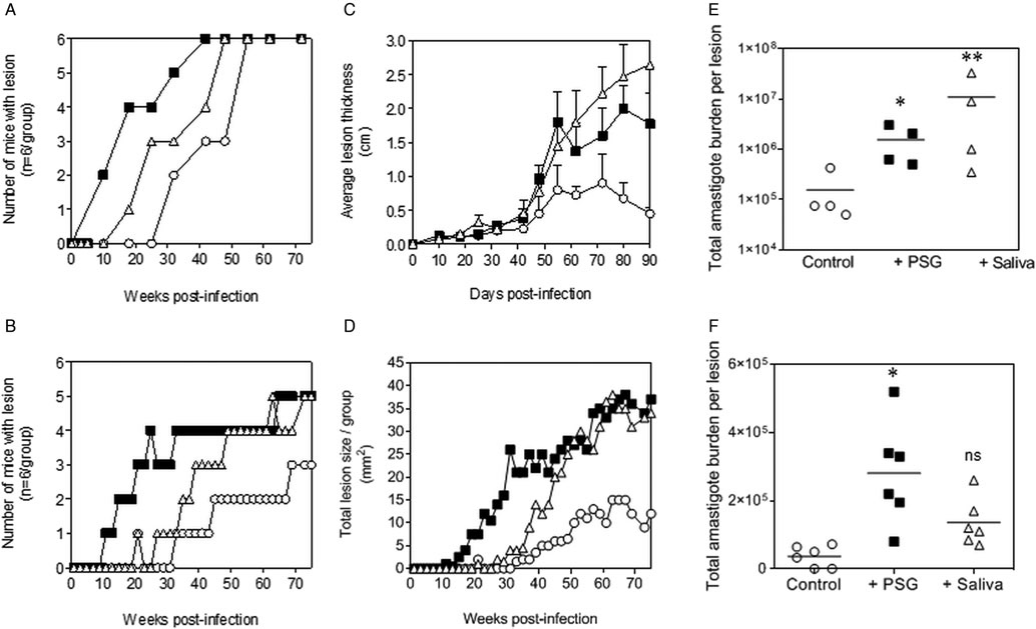
*L. major* and *L. tropica* infections in mice are exacerbated by PSG and saliva from the experimental vector, *Lu. Longipalpis (L. major:* A, C and E; *L. tropica*: B, D and F). *L. tropica* and *L. major* PSG was obtained from infected *Lu. longipalpis* sand flies and saliva collected from uninfected flies. One thousand *L. major* metacyclic promastigotes or one million *L. tropica* metacyclic promastigotes were injected i.d. into the footpads or rumps of BALB/c mice without (open circles) or with 1 *µ*g sterile PSG (closed squares) or with 1 *µ*g sterile saliva (open triangles). (A and B) Proportion of mice presenting with cutaneous lesions over the course of study. (C and D) Lesion evolution showing average lesion thickness + s.e.m or total lesion size. (E and F) Final parasite burdens of lesions from tissue homogenates. Each point represents individual mice and bars represent the average value per group. Representative data is shown of duplicate experiments, *n* = 6 mice per group. *: *P* < 0.05; **: *P* < 0.005 by Mann Whitney unpaired two-tailed *t*-test.

The PSG extracted from the experimental vector, *Lu. longipalpis* promoted *L. major* and *L. tropica* infection in BALB/c mice (Fig. 4). Similar to the infections using PSG from their natural vectors, the inclusion of *Lu. longipalpis*-derived PSG in the inoculum contracted the time to the first appearance of cutaneous lesions by 25 days and 21 weeks for *L. major* and *L. tropica*, respectively, compared to 35 days and 33 weeks for infections with metacyclic promastigotes alone (Fig. 4A and B). The resultant lesions also displayed accelerated growth (Fig. 4C and D) and higher final parasite burdens (Fig. 4E and F, *L. major*: 11-fold; *L. tropica*: 35-fold more amastigotes recovered from lesions), similar to those observed from the co-inoculation of PSG from their natural vectors. Saliva from *Lu. longipalpis* promoted cutaneous infection and pathology for *L. major* resulting in a 43-fold higher amastigote burden at the end of the experiment. For *L. tropica,* however, *Lu. longipalpis* saliva appeared to promote lesion size but had little influence over the growth of these parasites in skin (Fig. 4E and F). Collectively, these results show that PSG from *Lu. longipalpis* can exacerbate *L. major* and *L. tropica* to a similar extent as the material collected from their natural vectors.

## Discussion

This study demonstrates for the first time the infection-enhancing effect of PSG of two related CL *Leishmania* species from natural and unnatural phlebotomine sand fly vectors.

*L. major* is the causative agent of zoonotic cutaneous leishmaniasis, endemic in the Middle East and Northern Africa (Maroli *et al*., 2013). Its natural vectors include *P. papatasi* and *P. duboscqi* (Dvorak *et al*., 2018). Cutaneous infections in non-healing strains of mice, such as BALB/c, typically result in strong Th2 polarisation, driven largely by IL-4, allowing uncontrolled parasite growth (Noben-Trauth *et al*., 1996; Belkaid *et al*., 1998). Vector saliva benefits *L. major* infection in naïve hosts by biasing the local immune environment towards a Th2 phenotype, through increased expression of IL-4 and IL-10 (Belkaid *et al*., 1998). This immunomodulation promotes the initial infection and allows *L. major* to persist in skin, even after lesion resolution. The persistence of low numbers of *L. major* can establish concomitant immunity to reinfection and establish the host as a long-term reservoir of infection (Kimblin *et al*., 2008).

*L. tropica* overlaps in much of its range with *L. major* and is the causative agent of anthroponotic CL, endemic in the Middle East, Mediterranean basin, Central Asia, and East Africa. The primary vector of *L. tropica* is *Phlebotomus (Paraphlebotomus) sergenti* (Kamhawi *et al*., 2000; Myskova *et al*., 2007; Volf and Myskova, 2007), although other sand fly species have been shown to transmit, including *P. arabicus* in northern Israel (Jacobson *et al*., 2003; Svobodová *et al*., 2006*a*, 2006*b*) and Ethiopia (Gebre-Michael *et al*., 2004). *L. tropica* is responsible for cutaneous lesions which are typically drier, longer lasting, require longer to heal and are often more difficult to treat compared to *L. major*; resulting in considerable scarring (Klaus and Frankenburg, 1999). In mouse models, the persistence of *L. tropica* is also Th2-dependent, requiring manipulation of IL-10 and TGF-*β* signalling (Anderson *et al*., 2008; Kobets *et al*., 2012). Similar to *L. major*, chronic lesions of *L. tropica* in mice harbour sufficient numbers of parasites to be transmitted to their natural vector (Anderson *et al*., 2008).

Components of the infectious sand fly bite greatly influence the course of leishmaniasis in mammalian hosts (Serafim *et al*., 2017). This was first identified over thirty years ago, when it was found that the presence of *P. papatasi* sand fly saliva during *L. major* infection greatly promoted cutaneous lesion evolution and parasite growth (Titus and Ribeiro, 1988). However, sand fly saliva alone does not fully explain the efficiency or unique immune profile of *Leishmania* transmission (Serafim *et al*., 2017). Other factors, including the biting behaviour of the infected sand fly, the low dose delivered by bite, the wound damage associated with the sand fly bite and the presence of other parasite factors generated within the sand fly prior to transmission are likely to have a bearing on natural *Leishmania* infection. Understanding the contribution of these factors will allow us to generate more physiologically relevant models of *Leishmania* infection in the future. In this regard, we extend our previous findings with *L. mexicana* and *L. infantum* and show that the glycan-rich PSG from *L. major* and *L. tropica*, extracted from their natural vectors, can potently accelerate and exacerbate cutaneous infection in mice.

Using the experimental *L. mexicana*–*Lu. longipalpis* CL model of transmission we have previously shown that PSG promotes cutaneous infection by accelerating wound healing in skin of the mammalian host (Giraud *et al*., 2018). PSG recruited host macrophages to the site of infection and manipulated their physiology by upregulating the expression of arginase 1, an enzyme responsible for polyamine production and a signature of macrophage alternative (M2) activation (Rath *et al*., 2014). This effect appears to be under the control of insulin growth factor 1 pathway, as part of the wound healing process, resulting in the enhanced survival of *Leishmania* and their growth inside macrophages (Giraud *et al*., 2018). Using this parasite-vector model, we have also shown that the PSG-blockage impacts on the blood-feeding ability of the sand fly and promotes transmission by manipulating the sand fly feeding behaviour (Rogers and Bates, 2007). It is likely that both *L. major* and *L. tropica* would benefit from the ability of PSG to promote M2 macrophage activation, as polyamines are essential for their intracellular growth in mice (Green *et al*., 1990; Badirzadeh *et al*., 2017). Moreover, arginase 1 activity is closely associated with acute lesions of *L. major* and *L. tropica* in humans (Mortazavi *et al*., 2016).

Compared to *L. major, L. tropica* is a poorly studied parasite because of a lack of a reliable model of infection. In rats, *L. tropica* does not produce lesions despite surviving in the skin and being able to infect naïve sand flies for more than a year post-infection (Svobodová *et al*., 2003). In mice, although lesions can be generated, they are notoriously difficult and slow to establish. Lira *et al*., showed that experimental infections were possible in mice, requiring large doses (10^6^) of highly enriched metacyclic promastigotes (Lira *et al*., 1998). We extend their findings by revealing that both vector saliva and PSG from infected vectors can substantially accelerate their model of cutaneous *L. tropica* infection in mice, thereby highlighting the probable role of vector-derived products for the establishment of *L. tropica* infection and improving the current model of infection. In the future, it would be worth testing the exacerbatory role of PSG on *L. tropica* infection in the ear dermis, as this site of infection has been shown to produce reliable infections with lower numbers (10^3^–10^5^) of parasites (Anderson *et al*., 2008). In this study, it is striking that the growth of *L. tropica* in BALB/c mice appears to be much less vigorous than *L. major* but both species can benefit substantially from either the vector saliva or PSG. How this general mechanism of parasite survival contributes to *L. tropica* pathogenesis will require careful further study but is likely to be part of a complex of regulatory mechanisms, which subtly control both the Th1 and Th2 arms of the adaptive immune response.

PSG from natural *Leishmania*-sand fly combinations significantly contributes to the progression of the infection in skin of the mammalian host. From these findings, we can now add the parasite PSG to the list of vector-derived products that exacerbate *L. major* infection and significantly improve the currently intractable model of *L. tropica* infection (Kobets *et al*., 2012). Taken collectively, this reinforces the hypothesis that PSG is a common component of sand fly transmission and a virulence factor to *Leishmania* sand fly vector combinations found in nature. This has implications for interpreting the pathogenesis of *Leishmania* infection and the choice of an appropriate challenge for anti-leishmanial drug and vaccine screening.
